# Radiologically isolated aquaporin-4 antibody neuromyelitis optica spectrum disorder

**DOI:** 10.1177/13524585221074947

**Published:** 2022-03-25

**Authors:** Omar Abdel-Mannan, Ainat Klein, Anat Bachar Zipori, Liat Ben-Sira, Aviva Fattal-valevski, Yael Hacohen, Hadas Meirson

**Affiliations:** Queen Square MS Centre, UCL Queen Square Institute of Neurology, Faculty of Brain Sciences, University College London, London, UK; Department of Ophthalmology, Tel Aviv Sourasky Medical Center, Sackler Faculty of Medicine, Tel Aviv University, Tel Aviv, Israel; Department of Ophthalmology, Tel Aviv Sourasky Medical Center, Sackler Faculty of Medicine, Tel Aviv University, Tel Aviv, Israel; Department of Pediatric Radiology, Tel Aviv Sourasky Medical Center, Sackler Faculty of Medicine, Tel Aviv University, Tel Aviv, Israel; Department of Pediatric Neurology, Tel Aviv Sourasky Medical Center, Sackler Faculty of Medicine, Tel Aviv University, Tel Aviv, Israel; Queen Square MS Centre, UCL Queen Square Institute of Neurology, Faculty of Brain Sciences, University College London, London, UK; Department of Pediatric Neurology, Tel Aviv Sourasky Medical Center, Sackler Faculty of Medicine, Tel Aviv University, Tel Aviv, Israel

**Keywords:** Neuromyelitis optica spectrum disorder, aquaporin-4 antibody, paediatric demyelination, radiologically isolated syndrome

## Abstract

Aquaporin-4 antibody (AQP4-Ab) Neuromyelitis Optica Spectrum Disorder (NMOSD) is a rare neuroinflammatory syndrome presenting predominantly with optic neuritis and transverse myelitis. We report a case of radiologically isolated longitudinally extensive optic neuritis in an asymptomatic 12-year-old female with positive serum AQP4-Ab, with resolution of imaging changes after immune therapy. By contrast to patients with radiologically isolated syndrome, of which some will never convert to multiple sclerosis, the pathogenicity of AQP4-Ab in the context of sub-clinical disease, supported treatment in our patient. Given the severe morbidity in AQP4-Ab NMOSD, prognostic biomarkers for disease severity are required to guide optimal therapy for patients.

## Introduction

Aquaporin-4 antibody (AQP4-Ab) Neuromyelitis Optica Spectrum Disorder (NMOSD) is a rare neuroinflammatory syndrome presenting predominantly with optic neuritis and transverse myelitis. The discovery of AQP4-Ab in 2004 allowed expansion of the phenotype to include patients with single or recurrent attacks of optic neuritis, myelitis (longitudinally extensive or short) and brain/brainstem disease.^1^ AQP4-Ab is detected in the serum using cell-based assay with excellent sensitivity and specificity.^2^ Using the latest clinical diagnostic criteria, NMOSD can be diagnosed in the context a central nervous system (CNS) inflammatory event (especially with involvement of the optic nerve or spinal cord) and AQP-4-Ab positivity; seronegative patients require additional criteria to be met.^3^

The clinical features and magnetic resonance imaging (MRI) abnormalities in children with AQP4-Ab are similar to the adult phenotype. Although many children are left with visual or motor deficits after a single attack, children are reported to have less severe disease course, and may take longer to reach disability than adults with AQP4-Ab NMOSD. Nevertheless, the disease course appears more active with a higher annual relapse rate compared to adult-onset disease. In a large multi-centre study of 441 AQP4-Ab positive NMOSD adult and paediatric patients, mathematical modelling demonstrated that younger patients were more likely to have optic neuritis as an initial presentation, while older patients were more likely to have motor difficulties.^4^

Here, we report a case of radiologically isolated optic neuritis in a child who was found to have AQP4-Ab, despite not fulfilling 2015 NMOSD diagnostic criteria. Written informed consent for the publication of the case description and figure was obtained.

## Case report

**Figure 1.**
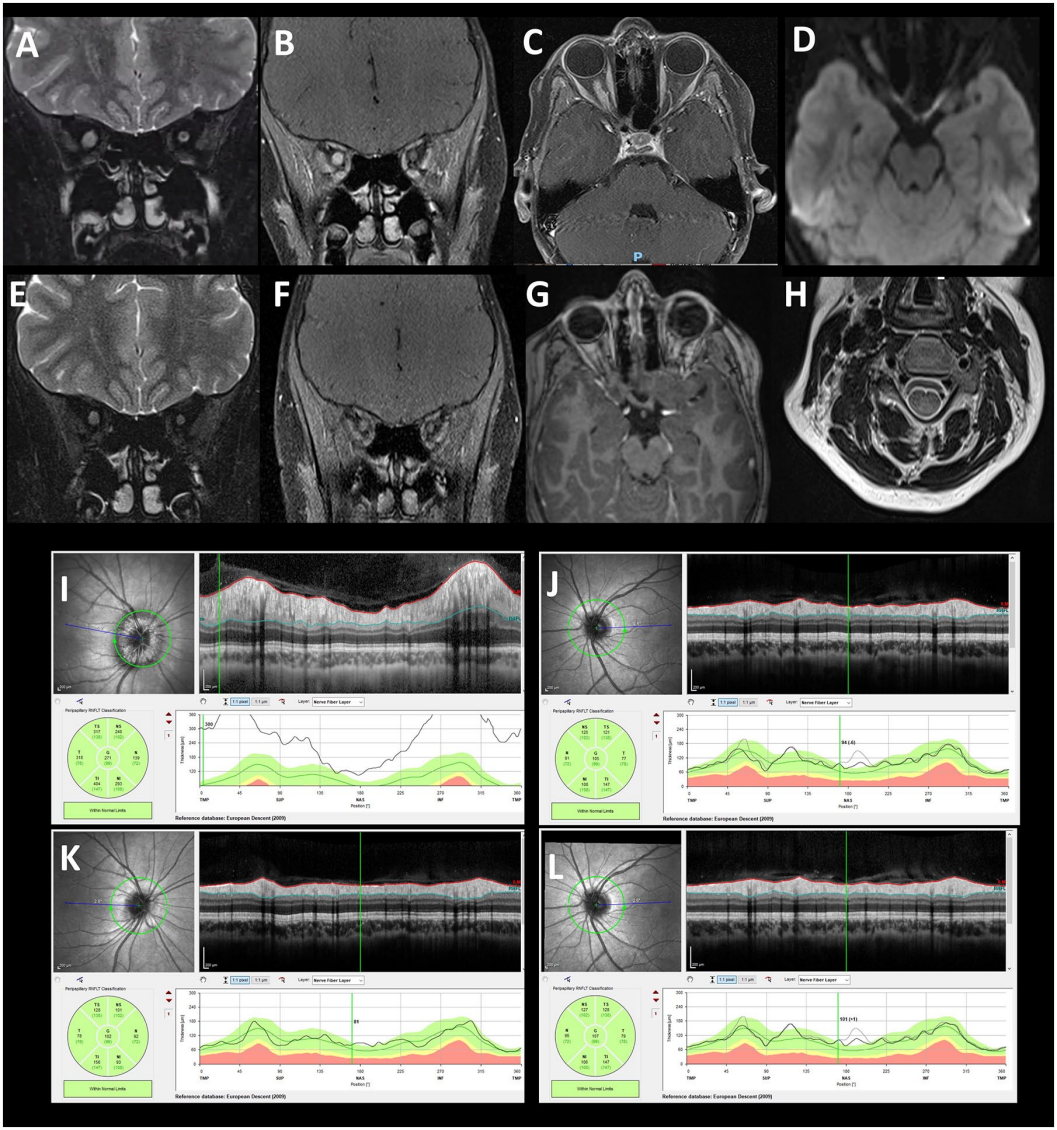
At presentation, coronal T2 imaging at onset demonstrating signal change (A) in the right optic nerve with gadolinium enhancement along the optic nerve along the infraorbital signal with sparing the optic chiasm (B) and (C). The right optic nerve also showed restricted diffusion (D). At 3-month follow-up, repeat neuroimaging showed resolution of the gadolinium enhancement previous changes with no new lesions (E), (F) and (G). An additional small non-enhancing central T2 hyperintense lesion at the level of C4 was also seen at presentation (H). Ophthalmologic assessment at presentation demonstrated oedema of the right optic nerve with normal visual fields. Retinal nerve fibre layer (RNFL) thickness on optical coherence tomography was increased (271 microns) on the right (I) and normal (108 microns) on the left eye (J). At 3-month follow-up, ophthalmologic assessment demonstrated improvement in optic disc swelling, with RNFL thickness on optical coherence tomography of 101 microns on the right (K) and normal as before (107 microns) on the left eye (L).

A previously healthy 12-year-old pre-pubertal female with no significant family history of auto-immune conditions was found to have right-sided optic disc swelling on routine eye examination at the opticians. She denied any headaches, vomiting, blurred vision, pain on eye movements or other neurological complaints. She had no focal neurology on examination. Ophthalmological assessment demonstrated right-sided optic disc swelling, with no haemorrhages, and no relative afferent pupillary defect. Visual acuity, colour vision and visual fields were normal. Retinal nerve fibre layer thickness on optical coherence tomography was 271 microns on the right and 108 microns on the left. Initial brain and spinal cord MRI demonstrated a longitudinally extensive optic neuritis with gadolinium enhancement (Figure 1). An additional small non-enhancing central T2 hyperintense lesion at the level of C4 was also seen. Cerebrospinal fluid analysis showed four white cells, normal protein, glucose and lactate concentrations, and no oligoclonal bands. Serum AQP4-Ab were positive on testing (cell-based assay), while serum myelin oligodendrocyte glycoprotein (MOG) antibodies were negative. Extensive screening for infectious and metabolic causes was also negative. She was treated with pulsed intravenous methylprednisolone (IVMP) 30 mg/kg for 5 days followed by one course of Rituximab (two doses of 750 mg/m^2^). She remained asymptomatic and ophthalmologic assessment at 3-month follow-up demonstrated improvement in optic disc swelling, with retinal nerve fibre layer thickness on optical coherence tomography of 101 microns on the right. In addition, repeat neuroimaging showed resolution of the previous changes with no new lesions (Figure 1).

## Discussion

We describe here a paediatric AQP4-Ab NMOSD patient who was clinically asymptomatic with normal visual acuity despite swollen optic disc and longitudinally extensive optic neuritis on imaging. Despite the patient not formally fulfilling the NMOSD diagnostic criteria, which requires at least one discrete clinical attack of CNS symptoms, it was felt by the clinicians that the potential risk of a clinical event justified pre-emptively treating with immune therapy. Another possible clinical approach would have been that of ‘watchful waiting’; that is, initiating treatment at the first sign of a clinical change, if this did in fact occur.

The majority of children with AQP4-Ab positive NMOSD relapse during their disease course, with higher disability score at 2 years from disease onset compared to multiple sclerosis. Children also suffer from higher visual disability compared to their adult counterparts, with optic neuritis presentation being associated with poor visual outcome.^5^ In a retrospective study of 37 patients (of which 27 were AQP4-Ab positive NMOSD), a delay in initial immune treatment with IVMP was detrimental to vision, highlighting the importance of early treatment for long-term visual recovery.^6^ The radiological resolution seen in our patient following the acute treatment may suggest that the early diagnosis and treatment prevented the incident clinical attack.

Interestingly, our patient had an asymptomatic spinal cord lesion in addition to the gadolinium enhancing optic nerve lesion. In AQP-4-Ab NMOSD, new lesions normally only occur in the context of a clinical event, although there have been recent isolated reports of both asymptomatic brain and spinal cord lesions in seropositive NMOSD,^7,8^ in addition to asymptomatic gadolinium enhancement of the optic nerves in the N-MOmentum clinical trial.^9^

In radiologically isolated syndrome (RIS), a term used to describe individuals with MS-like lesions without a clinical correlate, clinical signs may be present in the absence of a clinical event or neurological symptoms. In fact, there is no mention of clinical examination findings in formal diagnostic criteria. Given our patient had normal visual acuity, despite the presence of optic disc oedema noted on routine examination, and no focal neurology, we would use the same terminology here. By contrast to patients with RIS of which some will never convert to multiple sclerosis, the pathogenicity of AQP4-Ab in the context of sub-clinical disease (on neuroimaging and optical coherence tomography (OCT)), supported treatment in our patient. Our patient was treated with a course of Rituximab for relapse prevention following IVMP initially, and it remains challenging for clinicians to decide on the duration of immunosuppressive therapies for patients who have been in remission with no subsequent relapses. In fact, a study of 17 AQP4-Ab NMOSD showed that the discontinuation of immunosuppressive therapies may increase the risk of relapse in seropositive patients even after 5 years of remission.^10^ With the associated severe morbidity reported in AQP4-Ab NMOSD, prognostic biomarkers for disease severity are required to avoid both under- and over-treatment of these patients.
